# Aldehyde Dehydrogenase Polymorphisms and Blood Pressure Elevation in the Japanese: A Cross-Sectional and a Longitudinal Study over 20 Years in the Shimane CoHRE Study

**DOI:** 10.1155/2015/825435

**Published:** 2015-06-22

**Authors:** Minoru Isomura, Tao Wang, Masayuki Yamasaki, Md. Zahid Hasan, Kuninori Shiwaku, Toru Nabika

**Affiliations:** ^1^Department of Functional Pathology, Shimane University, Izumo 693-8501, Japan; ^2^The Center for Community-Based Health Research and Education (CoHRE), Shimane University, Izumo 693-8501, Japan; ^3^Department of Environmental and Preventive Medicine, School of Medicine, Shimane University, Izumo 693-8501, Japan

## Abstract

*Purpose*. Effects of a genetic polymorphism in the aldehyde dehydrogenase-2 (*ALDH2*) on blood pressure (BP) were investigated in a cross-sectional and a longitudinal study over 20 years on Japanese rural residents.* Methods*. Health examinations were held through 2006 to 2012, and 3,202 participates were recruited for this study. Among these participants, 560 individuals had medical records that were obtained in a health examination 20 years ago. Genomic DNA of participants was extracted from blood and the genotype of a polymorphism in* ALDH2* was determined by the TaqMan method. Multivariate regression analyses were performed to examine association between BP and the genetic polymorphism in the* ALDH2* gene.* Results*. Systolic and diastolic BP were higher in the *ALDH2* ^*∗*^ *1*/^*∗*^ *1* than the others (*ALDH2* ^*∗*^ *1*/^*∗*^ *2* or *ALDH2* ^*∗*^ *2*/^*∗*^ *2*). Genetic variation of the *ALDH2* gene apparently influenced drinking behavior as the number of the drinkers was significantly reduced in the *ALDH2* ^*∗*^ *2*/^*∗*^ *2* after 20 years of the observation period. This polymorphism, however, did not confer a risk for BP increase in the longitudinal observation. *Conclusion*. The present cross-sectional study confirmed a genetic effect of the *ALDH2* gene on BP. In contrast, no significant effects on BP were identified in a longitudinal study, which may require a careful consideration.

## 1. Introduction

Epidemiological studies have shown a significant relationship between the alcohol consumption and blood pressure (BP) [1–5]. It is conceivable that personal ability to metabolize ethanol (beverage alcohol) may modify such a relationship. The primary pathway of ethanol metabolism involves oxidation of ethanol to acetaldehyde, which is followed by oxidation to acetate catalyzed by aldehyde dehydrogenase-2 (*ALDH2: *NP_000681.2) [6].

A nonsynonymous polymorphism, Glu504Lys (rs671), is known to have large influence on the* ALDH2* activity [7]. This single nucleotide polymorphism (SNP) is frequently observed in Asians (~50% of the Japanese possess the minor allele, 504Lys), whereas it is rare in the Caucasians [8, 9]. The 504Lys allele, designated historically as* ALDH2* ^*∗*^ *2*, results in the enzymatically inactive form of* ALDH2* that fails to convert aldehyde into acetic acid rapidly. Accordingly, the concentration of serum acetaldehyde after alcohol intake reaches much higher levels in individuals with the* ALDH2* ^*∗*^ *2/* ^*∗*^ *2* and* ALDH2* ^*∗*^ *2/* ^*∗*^ *1 *genotype when compared with homozygote of the wild type allele,* ALDH2* ^*∗*^ *1/* ^*∗*^ *1* (19 and 6 times greater, resp.) [10, 11]. Since an elevated acetaldehyde level causes adverse reactions such as facial flushing, tachycardia, palpitations, and drowsiness in drinkers, even a small amount of alcohol consumption is often impossible for individuals with* ALDH2* ^*∗*^ *2/* ^*∗*^ *2 *[12, 13].

Considering these observations together, the* ALDH2* genotype was implicated as a factor modifying the adverse effect of drinking on BP [14–16]. In fact, a recent genome-wide association study (GWAS) on Asian populations indicated that the genetic locus including the* ALDH2* gene harbored a strong association with BP [17]. In this study, Kato et al. indicated that the* ALDH2* ^*∗*^ *2* allele associated with lower BP, which was highly dependent on drinking habit.

Accordingly, in this communication, we attempted to obtain further evidence supporting this result in a longitudinal study as well as in a cross-sectional study using a population in Japan.

## 2. Materials and Methods

### 2.1. Subjects

The study protocol was approved by the ethics committee of Shimane University School of Medicine and written informed consent was obtained from all participants.

Participants were recruited at health examinations held in Shimane prefecture from 2006 through 2012. In total, 3,202 participants were recruited. Measurements of height and weight were performed on site. Histories of smoking, alcohol consumption, diabetes mellitus, dyslipidemia, and medication were recorded at on-site interviews. Venous blood was collected after overnight fasting to measure biochemical parameters. Among those subjects, 560 received a health examination in 1986. This subgroup was used in a longitudinal assessment of the genetic effect of the* ALDH2* polymorphism on BP over 20 years. This subgroup was named the “longitudinal cohort.”

### 2.2. Blood Pressure Measurement

BP was measured in a sitting position at all health examinations. Measurements were performed by certified nurses using an automatic sphygmomanometer, after allowing subjects at least 15-minute sedentary rest. An average of 3 measurements was obtained. Hypertension is defined as either systolic BP (SBP) ≥140 mm Hg and/or diastolic BP (DBP) ≥90 mm Hg, or taking antihypertensive medication.

### 2.3. Genotyping

Genomic DNA was isolated from the peripheral blood samples. Genotyping of the Glu504Lys was performed by using the TaqMan method on a GeneAmp PCR 9700 machine (Thermo Fisher Scientific, Waltham, MA, USA) [18].

### 2.4. Statistical Analysis

Statistical analyses were performed with the R package (http://cran.r-project.org/). Differences in demographic data of subjects were analyzed by Student's *t*-test or *χ* ^2^ test, whichever was appropriate. Genotype frequencies and the Hardy-Weinberg equilibrium were estimated by the *χ* ^2^ test. Because the incidence of Lys/Lys was low (about 4.1%),* ALDH2* ^*∗*^2/^*∗*^2 and* ALDH2* ^*∗*^1/^*∗*^2 were combined together in the following analyses. Effects of the* ALDH2* genotype on BP were assessed by the multiple linear and the logistic regression analysis with adjustment for potential confounding factors including drinking habits.

## 3. Results

The frequency of the* ALDH2* genotypes in the studied population (0.61, 0.35, and 0.04 for ^*∗*^1/^*∗*^1, ^*∗*^1/^*∗*^2, or ^*∗*^2/^*∗*^2, resp.) was in agreement with those previously observed in the Japanese [7, 10] and was within the Hardy-Weinberg equilibrium.

Association between the* ALDH2* polymorphism and BP was examined first in a cross-sectional analysis.  Table 1 shows the demographic characteristics of the subjects. There was no significant difference between the two genotype groups in regard to gender composition, age, body mass index (BMI), and drinking habit. SBP and DBP were greater in* ALDH2* ^*∗*^1/^*∗*^1 than the other genotypes although the genetic effect of the genotype on BP was attenuated when adjusted with drinking habit (see Table 1).  Table 2 summarizes odds ratio of risk factors for hypertension calculated by the logistic regression analysis; the genotype of the Glu504Lys polymorphism was an independent risk factor for hypertension even after being adjusted with confounding factors including drinking habit.

We then examined the effect of the* ALDH2* polymorphism on BP change in the longitudinal cohort.  Table 3 shows the demographic characteristics of the subjects of the cohort. SBP was significantly elevated after 20 years of observation, whereas only a marginal change was found in DBP. Because elevation of SBP was significantly greater in women than in men, the following analysis was performed on both genders separately. In 2006, the number of habitual drinkers was reduced significantly compared to that in 1986. No significant effects of the* ALDH2* genotype were identified either on the change in SBP or in DBP (Table 4); even analyses were performed separately on drinking habits in 1986 and/or 2006 (data not shown). Multivariate analysis indicated that only ΔBMI in men showed a significant association with ΔSBP (see Table S2 of the Supplementary Material available online at http://dx.doi.org/10.1155/2015/825435).

## 4. Discussion

The present study confirmed that the Glu504Lys polymorphism in the* ALDH2* gene had a significant effect on BP under a cross-sectional study design.

A recent result of GWAS indicated that the Glu504Lys (rs671) was associated with BP in Asians including the Japanese [17]. According to this report, individuals carrying the G allele (*ALDH2* ^*∗*^1) showed significantly higher BP than those carrying the A allele (*ALDH2* ^*∗*^2). Of note, this association was largely influenced by drinking habit. This was observed also in our cross-sectional study. Habitual alcohol drinking has been demonstrated as a strong risk factor for hypertension [2, 3]. As individuals with* ALDH2* ^*∗*^1/^*∗*^1 are expected to consume more alcohol than those with the other genotypes, most part of the association of the* ALDH2* polymorphism with hypertension might be due to difference in alcohol intake. However, our subgroup analysis revealed that the* ALDH2* polymorphism was a risk factor for hypertension even in nonhabitual drinkers (see Table S1). This result implies that the* ALDH2* genotype has an additional effect on BP independently of the alcohol intake. In addition to the dehydrogenase activity, ALDH shows a wide variety of biological activities, which related to oxidative stress, inflammation, and the serum level of asymmetric dimethylarginine (ADMA) [19]. However, it is unclear how the differences in genotypes of* ALDH2* gene affect those activities that lead to the difference in blood pressure. Physiological and/or biochemical studies are essential to evaluate effects of the SNP on the metabolic pathway. In addition, a comprehensive genetic analysis in the* ALDH2* locus may be necessary to identify polymorphisms responsible for this “alcohol-independent” effect because Glu504Lys may be a “passenger” polymorphism merely linked with the true responsible polymorphisms [17, 20].

Although the association between the* ALDH2* genotype and BP was confirmed in a cross-sectional analysis, it was not replicated in a longitudinal study. There are several possible reasons for this discrepancy; first of all, due to a small number of participants in the longitudinal study, the statistical power was not enough to show the effect of the* ALDH2* genotype.

Second, age of participants in the longitudinal study was too high at the start of the study. In 1986, the mean age of participants was 51 years which was rather too old to study the development of hypertension longitudinally. Indeed, 319 of 560 individuals (56%) already had hypertension at that time. At this age, many of the participants harbored multiple risk factors for hypertension such as arteriosclerosis, even if they did not develop overt hypertension yet. This might have masked the effects of the genetic risk factors. Supporting this interpretation, age and drinking status, both of which are established risk factors for hypertension, failed to show a significant association with ΔSBP in the present longitudinal study. Further, menopause might be an additional disturbing factor on BP change among women [21, 22]; most of the female subjects in the study were around the age of menopause in 1986, and they might show greater increase in BP during observational 20 years.

Third, in our study population, one-third of daily drinkers in 1986 quitted drinking during the following 20 years. Change in drinking habit might influence the genetic effect of the* ALDH2* polymorphism.

In conclusion, we confirmed the effect of the Glu504Lys polymorphism in the* ALDH2* gene on BP in a cross-sectional study, though we failed to show the association in a longitudinal analysis. Further studies using a large number of younger individuals are essential to obtain a conclusive result on the effect of the* ALDH2* genotype on a longitudinal change of BP.

## Supplementary Material

Table S1. The risk of the ALDH2 gene polymorphism on hypertension was examined separately by drinking status. These analyses revealed that the ALDH2 polymorphism was a risk factor for hypertension even in non-habitual drinkers.
Table S2. Multivariate linear regression analysis on factors influencing ΔSBP in the longitudinal study showed that that only ΔBMI in men showed a significant association with ΔSBP.

## Figures and Tables

**Table 1:** Demographic data of subjects with different *ALDH2* genotypes.

	*ALDH2* genotype		*p* value adjusted with drinking habit	*p* value adjusted without drinking habit
	*ALDH2* ^*∗*^1/^*∗*^1	*ALDH2* ^*∗*^1/^*∗*^2 or *ALDH2* ^*∗*^2/^*∗*^2		
*N* (M/F)	1947 (864/1083)	1255 (566/689)	n/s	n/s
Age	65.9 ± 0.2	66.7 ± 0.3	n/s	n/s
BMI	22.6 ± 0.06	22.5 ± 0.08	n/s	n/s
SBP	134.0 ± 0.6	132.2 ± 0.6	0.004	0.0002
DBP	81.6 ± 0.3	80.3 ± 0.4	0.001	8.8 × 10^−6^
Daily drinker	654 (33.6%)	188 (15.0%)	2.2 × 10^−16^	—

*N*: number of individuals, M: male, F: female, and n/s: not significant.

**Table 2:** Odds ratios of risk factors for hypertension.

Risk factor	Odds (95% C.I.)	*p* value
Age	1.07 (1.06–1.08)	2 × 10^−16^
BMI	1.22 (1.19–1.26)	2 × 10^−16^
Daily drinker	1.45 (1.73–1.78)	0.00058
*ALDH2* ^*∗*^1/^*∗*^1	1.25 (1.06–1.46)	0.007

C.I.: confidence interval.

Hypertension is defined as SBP ≥ 140 mm Hg and/or DBP ≥ 90 mm Hg or taking antihypertensive medication.

**Table 3:** Changes of characteristics in parameters over 20 years in the longitudinal study.

	Years investigated		*p* value
	1986	2006	
*N*			
Male	212		
Female	348		
Age			
Male	49.2 ± 0.7	69.2 ± 0.7	
Female	50.2 ± 0.5	70.7 ± 0.5	
SBP			
Male	126.7 ± 1.4	132.7 ± 1.2	0.001
Female	121.9 ± 1.2	136.2 ± 1.1	2.2 × 10^−16^
DBP			
Male	82.7 ± 0.9	80.1 ± 0.7	0.03
Female	76.6 ± 0.6	78.1 ± 0.5	n/s
BMI			
Male	22.8 ± 0.2	22.8 ± 0.2	n/s
Female	22.9 ± 0.2	22.5 ± 0.2	n/s
Daily drinker			
Male	154	103	2.5 × 10^−5^
Female	10	11	n/s

**Table 4:** Association analysis between the BP change and the *ALDH2* genotypes.

	*ALDH2* genotype		*p* value
	*ALDH2* ^*∗*^1/^*∗*^1	*ALDH2* ^*∗*^1/^*∗*^2 or *ALDH2* ^*∗*^2/^*∗*^2	
*N* (M/F)	339 (119/210)	231 (93/138)	n/s
ΔSBP	11.0 ± 0.2	12.0 ± 1.1	n/s
ΔDBP	1.0 ± 0.7	1.0 ± 0.8	n/s

ΔSBP and ΔDBP indicate values of increase in systolic blood pressure or diastolic blood pressure, respectively: change in SBP and DBP after 20 years of the observation period. n/s: not significant.
