# Supplementary material for: Aldehyde Dehydrogenase Polymorphisms and Blood Pressure Elevation in the Japanese: A Cross-Sectional and a Longitudinal Study over 20 Years in the Shimane CoHRE Study
Source: Dis Markers. 2015 Jun 22;2015:825435. doi: 10.1155/2015/825435 (PMC4491569; doi:10.1155/2015/825435)
Supplement: Supplementary file 1 — Table S1. The risk of the ALDH2 gene polymorphism on hypertension was examined separately by drinking status. These analyses revealed that the ALDH2 polymorphism was a risk factor for hypertension even in non-habitual drinkers. Table S2. Multivariate linear regression analysis on factors influencing ΔSBP in the longitudinal study showed that that only ΔBMI in men showed a significant association with ΔSBP. [file 825435.f1.pdf]

Table S1. Evaluation of risks for hypertension in daily drinkers and in occasional/non drinkers

1) Daily drinkers

Risk factor	Odds (95% C.I.)	p value
Age	1.06 (1.04 - 1.08)	$2x10^{-15}$
BMI	1.23 (1.17 - 1.30)	$7x10^{-14}$
ALDH2 Glu/Glu	1.28 (0.91 - 1.83)	0.16

2) Occasional/non drinkers

Risk factor	Odds (95% C.I.)	p value
Age	1.08 (1.07 - 1.09)	$2x10^{-16}$
BMI	1.21 (1.18 - 1.26)	$2x10^{-16}$
ALDH2 Glu/Glu	1.27 (1.06 - 1.52)	0.008

Table S2. Linear regression analysis on factors influencing ΔSBP in the longitudinal study

Male

	Estimate β	Std. Error	p value
(Intercept)	1.13575	9.30957	n/s (0.9)
*ALDH2**1/*1	1.91000	2.73191	n/s (0.49)
age	0.04288	0.12771	n/s (0.74)
ΔBMI	2.68919	0.66613	**$7.8x10^{-5}$**
non-drinker	3.28719	2.69969	n/s (0.23)

Female

	Estimate β	Std. Error	p value
(Intercept)	14.11211	13.00460	n/s (0.28)
*ALDH2**1/*1	-3.57603	2.03483	n/s (0.07)
age	0.02461	0.10136	n/s (0.81)
ΔBMI	0.72083	0.47035	n/s (0.13)
non-drinker	0.86587	10.74908	n/s (0.94)

ΔBMI indicates change in BMI between 1987 and 2006. In this analysis, 'non-drinker' was defined as an individual who was non-drinker both in 1987 and in 2006. n/s: not significant.
